# Exploring the Utility of the Modified Hospitalized‐Patient One‐Year Mortality Risk Score to Trigger Referrals to Palliative Care for Inpatients With Cancer

**DOI:** 10.1002/cam4.70292

**Published:** 2024-10-09

**Authors:** A. Ghoshal, R. Prince, J. Downar, J. Lapenskie, S. Subramaniam, P. Wegier, L. W. Le, B. Hannon

**Affiliations:** ^1^ Princess Margaret Cancer Centre Toronto Ontario Canada; ^2^ Department of Medicine, Division of Palliative Medicine University of Toronto Toronto Ontario Canada; ^3^ Epworth Cancer Services Clinical Institute Geelong Victoria Australia; ^4^ Department of Medicine, Division of Palliative Care University of Ottawa Ottawa Ontario Canada; ^5^ Ottawa Hospital Research Institute and Bruyère Research Institute Ottawa Ontario Canada; ^6^ Humber River Health North York Ontario Canada; ^7^ Department of Biostatistics Princess Margaret Cancer Centre Toronto Ontario Canada

**Keywords:** clinical decision‐making, palliative care, prognosis, scoring, supportive care

## Abstract

**Background:**

Estimating prognosis can be a barrier to timely palliative care involvement. The modified Hospitalized‐patient One‐year Mortality Risk (mHOMR) score uses hospital admission data to calculate the risk of death within 12 months and may be a useful tool to trigger a referral to palliative care.

**Methods:**

The mHOMR tool was retrospectively applied to consecutive acute admissions to a quaternary cancer center in Toronto, Canada from March 1 to May 31, 2018. The study aimed to investigate the association between dichotomized mHOMR scores (the cohort median score of 0.27 and the developer‐recommended score of 0.21) and the risk of death, and whether these could be used to identify patients who may benefit from timely palliative care involvement.

**Results:**

Of 269 inpatients, 87 were elective admissions and excluded from further analyses. At the median mHOMR score of 0.27, 91/182 patients (50%) were categorized as high‐risk of death within 12 months (mHOMR+), 53 (58%) were referred to palliative care. At the lower cut‐off of 0.21, 103 patients were mHOMR+, of whom 57 (55.3%) were referred to palliative care. The higher mHOMR was significantly associated with mortality (29.7% mHOMR− vs. 39.8% mHOMR+ at 12 months, log‐rank *p* < 0.05). The association between the developer‐recommended mHOMR cut‐off (≥ 0.21) and mortality was not significant (*p* = 0.15).

**Conclusions:**

A higher mHOMR score was significantly associated with the risk of mortality in patients with advanced cancer. However, the developer‐recommended mHOMR cut‐off of 0.21 failed to identify a statistically significant difference between patients with advanced cancer at low versus high scores. While mHOMR may be a useful tool to augment clinical judgment and identify inpatients with advanced cancer at high risk of death, who in turn may benefit from referral to palliative care, the optimal mHOMR cutoff may warrant adjustment for this population.

## Graphical abstract

The Modified Hospitalized‐patient One‐year Mortality Risk (mHOMR) score is an automated tool that captures patient demographics, health burden, and illness severity at the time of acute admission to predict the one‐year mortality risk of hospitalized patients. In our study, we retrospectively applied to mHOMR tool to a cohort of patients admitted to a tertiary cancer center. Our study provides valuable insights into the potential of the mHOMR score as a tool for identifying inpatients with cancer who may benefit from a referral to palliative care and suggests that the recommended cut‐off threshold may vary based on the patient population.

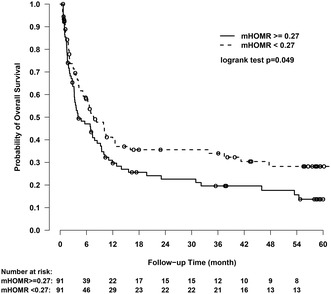

## 1 Introduction

Over the past several decades, there has been an increase in both the incidence and prevalence of cancer worldwide [1]. The rise in cancer cases has been increasingly met with the development of treatment options for advanced‐stage disease, including novel chemotherapies, targeted therapies, immunotherapy, precision medicine, and advances in surgical techniques [2]. The pursuit of aggressive therapies towards the end of life can result in delayed advance care planning, goals of care discussions, and goal‐incongruent care [3, 4, 5]. Early integration of palliative care alongside oncology services for patients with advanced cancer has been shown to improve symptom burden, quality of life, and satisfaction with care, as well as to reduce the aggressiveness of care at the end of life, and is now endorsed by multiple international cancer organizations [6, 7, 8].

Despite evidence supporting early palliative care, barriers to engagement persist, including an international shortage of specialist providers and variable referral patterns to palliative care services [9]. Standardized referral criteria based on disease stage, symptom burden, or physicians' best estimation of prognosis have been suggested, but are variably used [10, 11]. Automated applications using data collected routinely on hospital admission may help identify patients at risk of death, prompting referral to inpatient palliative care services [12]. One such tool is the modified Hospitalized‐patient One‐year Mortality Risk score (mHOMR), which employs nine data parameters available electronically upon admission to compute the mortality risk for the ensuing 12 months. mHOMR was designed to prompt clinical teams to consider appropriate interventions for patients with an increased mortality risk. The feasibility and acceptability of mHOMR have been previously demonstrated; it has been shown to exhibit greater accuracy compared to other existing prognostic tools relying on clinical information or clinician judgment. While the developers suggest a cut‐off score of 0.21, they recognize alternative cut‐offs may be preferable, based on the patient population and institutional resources [13, 14, 15].

We aimed to retrospectively apply mHOMR to a cohort of patients with advanced cancer admitted acutely, and to explore the association between mHOMR score and risk of mortality, to identify patients who may benefit from referral to an inpatient palliative care team.

## 2 Methods

### 2.1 Context

The Princess Margaret Cancer Centre (PM) is the largest comprehensive cancer center in Canada with 120 inpatient beds. The palliative care program consists of a 12‐bed inpatient palliative care unit, an outpatient program, and an inpatient consultation service. Currently, patients admitted under oncology services are referred to the inpatient palliative care consultation service at the discretion of their primary team of physicians.

### 2.2 Design

A retrospective cohort study, where consecutive non‐elective oncology adult admissions to PM from March 1 to May 31, 2018, were reviewed. This period was chosen to allow for long‐term follow‐up to death.

### 2.3 Exposure

Consistent with previous studies [13], we divided the patients into two groups (mHOMR positive vs. negative) based on their mHOMR score using two different cut‐offs: (1) the median mHOMR score for our cohort, which was 0.27. The median value dichotomizes the mHOMR score, guaranteeing an equal sample size for both groups; and (2) the mHOMR score of 0.21 recommended by the developers based on a previous study involving 400 inpatients with frailty, chronic organ failure, and cancer.

### 2.4 Data Collection

We extracted the nine pre‐defined mHOMR data points for each patient from the electronic medical record: age; sex; living status (home, long‐term care facility, rehabilitation, chronic care facility); admitting service; admission within the previous 30 days; number of Emergency Department visits within the previous 12 months; number of admission via ambulance within the previous 12 months; urgency of admission; and whether the patient was admitted directly to the Intensive Care Unit. We also collected cancer diagnosis and stage, code status on admission; hospital length of stay, date of death (if available) or date of last clinical contact, and whether the patient was seen by the palliative care team during admission (if not, whether they were seen by a palliative care team before death or date of last contact).

### 2.5 Analysis

Data were summarized as frequency and percentage for categorical variables and mean with standard deviation or median with range for continuous variables. Chi‐Square and Wilcoxon rank‐sum tests were used for the comparison of categorical and continuous variables, respectively. The Kaplan–Meier estimator was used to estimate the overall survival from the day of admission to the date of death or censored at the last known alive date if survival status was unknown. The log‐rank test was used to explore the difference in overall survival between groups. All analyses were conducted using open‐source software, RStudio (version 2023.06.1 + 524). All statistical assessments were 2‐sided; *p* < 0.05 was considered statistically significant.

## 3 Results

**FIGURE 1:**
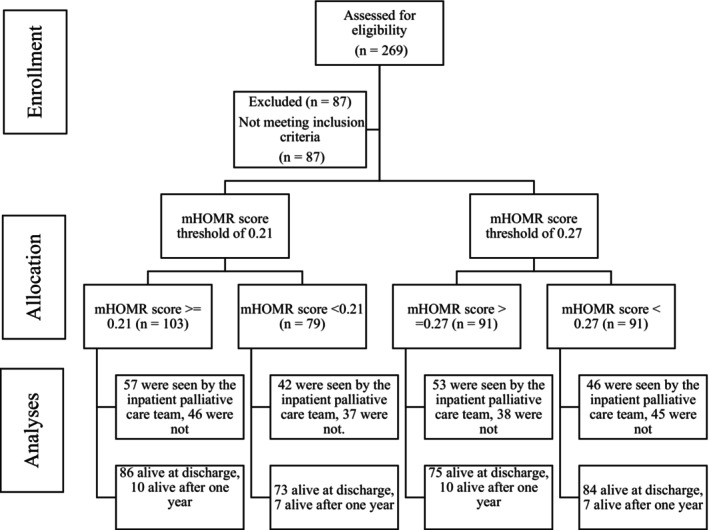
CONSORT diagram for observational study.

During the study period, a total of 269 patients were admitted, with 87 (32%) admitted for elective procedures (e.g., gastrostomy tube insertion or brachytherapy) and excluded from further analyses (see Figure 1). Among the remaining 182 patients, 89 (49%) were female, with an average age of 62 years, a mean length of stay of 14 days, and a median mHOMR score of 0.27 (Table 1). The predominant primary cancer sites were gastrointestinal (21%) and lung (20%); 82% had stage IV disease. The primary reasons for admission were symptom management (64%) and suspected infection or sepsis (23%). Most patients were living independently at home prior to admission (98%), and a sizable proportion of admissions were non‐emergent (64.8%). Upon admission, 125 (68%) had indicated their preference for ‘full code’ status, signifying a desire for aggressive medical interventions, including cardiopulmonary resuscitation (CPR), intubation, and mechanical ventilation, if necessary. Ultimately, 159 (87%) patients were discharged alive, and 99 (54%) were seen by the palliative care team during their hospitalization.

**TABLE 1:** Demographic and clinical features of participating patients, *N* = 182.

Characteristics	Number (%)	
Sex		
Men	93 (51)	
Women	89 (49)	
Admitting service		
Medical oncology	135 (74)	
Radiation oncology	47 (26)	
Cancer site		
Gastrointestinal	38 (20.9)	
Thoracic	36 (19.8)	
Gynecological	34 (18.7)	
Head neck	22 (12.1)	
Genitourinary	17 (9.3)	
Others	35 (19.2)	
Stage		
I and II	7 (4.2)	
III	9 (5.4)	
IV	150 (90.4)	
Unknown	16	
Living status		
Independent at home	179 (98.3)	
Care at home	2 (1.1)	
Chronic care hospital	1 (0.5)	
Urgency of admission		
Elective/planned admission*	118 (64.8)	
Emergency department via ambulance	15 (8.2)	
Emergency department without an ambulance	49 (26.9)	
Discharged alive from the hospital	159 (87.4)	
	Mean (SD)	Median (range)
Number of emergency visits in the past year	1 (2.5)	0 (0‐23)
Number of visits by ambulance in the past year	0.2 (0.55)	0 (0‐4)
Age (in years)	62 (14.2)	63 (19‐92)
Hospital length of stay (days)	14.4 (15.1)	9 (0.2‐278)
mHOMR score	0.32 (0.23)	0.27 (0.02‐0.90)

* Including admissions from outpatient oncology clinics.

Based on a median mHOMR score of 0.27, 50% of the 182 patients in this cohort were classified as mHOMR positive (mHOMR+). A comparison between mHOMR+ and mHOMR− patients revealed that mHOMR+ patients were more likely to be male (63% vs. 39%), had a slightly higher average number of emergency visits in the preceding 12 months (1.24 vs. 1.22, *p* < 0.05), were less likely to have a ‘full code’ designation at admission (66% vs. 71%, *p* < 0.05), and were less likely to have been admitted non‐emergently (79% vs. 81%). Of the 91 mHOMR+ patients, 53 (58%) were seen by the inpatient palliative care team during their admission. A comparison between mHOMR+ patients seen by palliative care and those not seen revealed that those seen by palliative care were slightly older (mean age 63 vs. 62 years, *p* < 0.05), less likely to have a ‘full code’ designation (57% vs. 79%), less likely to be discharged alive (75% vs. 92%), and less likely to be alive 12 months after the admission (4% vs. 21%). Additionally, 46 (50%) of mHOMR− patients were seen by the palliative care team.

Utilizing an mHOMR score of 0.21, 56% of the patients were mHOMR+. A comparison between mHOMR+ and mHOMR− patients with this score showed that mHOMR+ patients were older (mean age 69 vs. 52 years, *p* < 0.05), more likely to be male (62% vs. 36.7%), had a higher mean number of emergency visits in the preceding 12 months (1.57 vs. 0.73, *p* < 0.05), and were less likely to have been admitted non‐emergently (43.7% vs. 92.4%, *p* < 0.05). The mHOMR+ patients also had a longer mean length of stay (19 vs. 11 days, *p* < 0.05). Among the mHOMR+ cohort, 57 (55.3%) were seen by the inpatient palliative care team. A comparison between mHOMR+ patients seen by palliative care and those not seen revealed that those seen had a higher mean mHOMR score (0.53 vs. 0.42, *p* < 0.05), were younger (mean age 67 vs. 72 years, *p* < 0.05), less likely to have a “full code” designation at admission (57% vs. 82.6%, *p* < 0.05), and less likely to be discharged alive (77.2% vs. 91.3%, *p* < 0.05). Moreover, 42 (53%) of mHOMR− patients were seen by the palliative care team.

**FIGURE 2:**
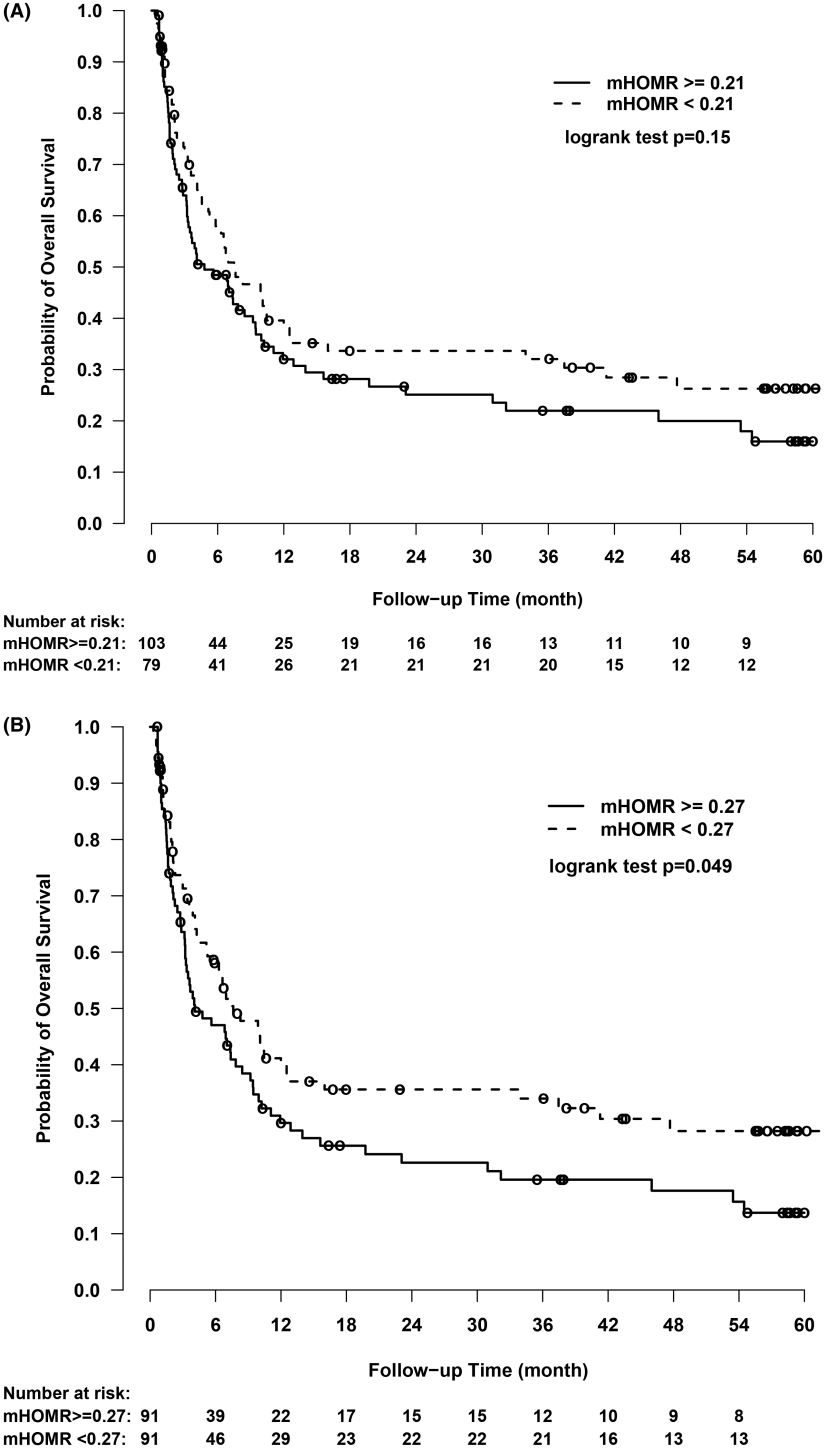
Kaplan–Meier plots. (A) For mHOMR score 0.27 (median). (B) For mHOMR score 0.21.

Utilizing the mHOMR score of 0.27, there was a statistically significant difference in survival probability between mHOMR+ (29.7% [95% CI: 21.3%–41.3%] at 1 year and 22.6% [95% CI: 15.0%–34.0%] at 2 years) and mHOMR− patients (39.8% [95% CI: 30.4%–52.1%] at 1 year and 35.6% [95% CI: 26.5%–47.9%] at 2 years); Chisq = 3.88 on 1° of freedom, log‐rank *p* = 0.049 on comparing the entire survival curves, see Figure 2A. There was no significant difference in cumulative survival probability between those patients with low versus high mHOMR scores when set at 0.21, *p* = 0.15 (Figure 2B).

## 4 Discussion

Our study demonstrates a significant association between mHOMR and 12‐month mortality risk for a group of hospitalized patients with advanced cancer using the cohort median mHOMR score of 0.27; this association was not significant when the developer‐recommended mHOMR cut‐off score of 0.21 was applied. Our findings suggest that mHOMR may be a useful tool to help identify a subset of hospitalized patients with advanced cancer who are at high risk of death within 12 months and who may, in turn, benefit from referral to palliative care services (Table 2). However, the developer‐recommended cut‐off of 0.21 may be inappropriate for this population and the optimal cut‐off warrants further exploration.

**TABLE 2:** Comparison of patients according to mHOMR status and referral to palliative care (*N* = 182).

Clinical items	mHOMR+				mHOMR−				
	*n* = 91	Referred to palliative care			*n* = 91	Referred to palliative care			
		Yes (*n* = 53)	No (*n* = 38)	*p*		Yes (*n* = 46)	No (*n* = 45)	*p*	*p (mHOMR+ vs. mHOMR−)*
(A) by median mHOMR score (≥ 0.27)									
Median mHOMR score	0.28	0.28	0.28	NA	0.26	0.26	0.26	NA	< 0.05
Average length of stay (in days)	16	16	16	NA	15.9	16	16	NA	NA
Code status at admission									
Full code	60	30	30	0.58	65	33	32	0.97	< 0.05
DNR/I	31	23	8		26	13	13		
Cancer diagnosis (site)									
Gastrointestinal	18	11	7	0.79	20	13	7	0.14	0.73
Thoracic	20	10	10	0.40	16	8	8	0.96	0.45
Gynecological	14	11	3	0.09	20	9	11	0.58	0.26
Head neck	8	3	5	0.21	11	5	6	0.73	0.47
Genitourinary	13	7	6	0.73	3	1	2	0.56	0.09
Others	18	11	7	0.79	21	10	11	0.76	0.58
Cancer diagnosis (stage)									
I and II	10	0	10	NA	9	4	5	NA	
III	4	1	3		6	0	6		
IV	75	51	24		75	42	33		
Others	2	1	1		1	0	0		
Admissions for symptom management	53	36	17	0.52	47	31	16	0.96	1.0
Discharged alive from the hospital	75	40	35	0.75	84	41	43	0.99	1.0
Alive after 1 year	10	2	8	0.81	7	3	4	0.99	1.0
mHOMR items									
Mean age (in years)	62	63	62	NA	62	62	62	NA	NA
Sex									
Male	57	30	27	0.48	36	18	18	0.95	1.0
Female	34	23	11		55	28	27		
Average number of hospital admissions via ambulance in the last 12 months	0.19	0.19	0.19	NA	0.19	0.19	0.19	NA	NA
Average number of emergency visits in the last 12 months	1.24	1.25	1.23	0.11	1.22	1.22	1.22	NA	< 0.05
Urgent re‐admission within ≤ 30 days of previous admission									
Yes	19	10	9	0.73	17	7	10	0.98	1.0
No	72	43	29		74	39	35		
Admitting service									
Medical oncology	68	44	24	0.72	67	38	29	0.98	1.0
Radiation oncology	23	9	14		24	8	16		
Living status before hospital admission									
Independent	89	52	37	NA	90	46	44	NA	NA
At home with care	1	1	0		1	0	1		
In a rehabilitation facility	0	0	0		0	0	0		
In a long‐term care facility	0	0	0		0	0	0		
In a chronic care facility	1	0	1		0	0	0		
Direct admission to the intensive care unit									
Yes	1	0	1	NA	1	0	1	NA	NA
No	90	53	37		90	46	44		
Survival to discharge									
Yes	75	40	35	0.75	84	41	43	0.65	1.0
No	16	13	3		7	5	2		
Urgency of admission									
Elective	34	12	22	0.59	84	42	42	NA	1.0
Emergency department visit via ambulance	14	9	5		1	1	0		
Emergency department visit without ambulance (including admissions from clinic)	43	32	11		6	3	3		

	mHOMR +				mHOMR−				
	*n* = 103	Referred to Palliative care			*n* = 79	Referred to Palliative care			
Clinical items		Yes (*n* = 57)	No (*n* = 46)	*p*		Yes (*n* = 42)	No (*n* = 37)	*p*	*p (mHOMR+ vs. mHOMR−)*
(B) By mHOMR score of 0.21									
Mean mHOMR score	0.48	0.53	0.43	< 0.05	0.12	0.11	0.12	0.78	< 0.05
Average length of stay (in days)	19	21	17	0.56	11	12	10	0.45	< 0.05
Code status at admission									
Full code	71	33	38	< 0.05	54	23	31	< 0.05	0.93
DNR/I	32	24	8		25	19	6		
Cancer diagnosis (site)									
Gastrointestinal	24	14	10	0.73	14	10	4	0.13	0.36
Thoracic	22	11	11	0.57	14	7	7	0.79	0.55
Gynecological	15	11	4	0.13	19	9	10	0.56	0.10
Head neck	11	3	8	0.05	11	5	6	0.58	0.51
Genitourinary	14	7	7	0.67	3	2	1	0.63	0.20
Others	17	11	6	0.39	18	9	9	0.76	0.29
Cancer diagnosis (stage)									
I and II	6	0	6	NA	8	2	6	NA	NA
III	9	1	8		7	2	5		
IV	86	54	32		64	38	26		
Others	2	2	0		0	0	0		
Admissions for symptom management	67	35	32	0.39	49	25	24	0.62	0.67
Discharged alive from the hospital	86	44	42	0.05	73	37	36	< 0.05	0.07
mHOMR items									
Mean age (in years)	69	67	72	< 0.05	52	62	53	0.58	< 0.05
Sex									
Male	64	32	32	0.16	29	16	13	0.78	< 0.05
Female	39	25	14		50	26	24		
The average number of hospital admissions via ambulance in the last 12 months	0.31	0.35	0.26	0.51	0.02	0.02	0.02	00.93	< 0.05
Average number of emergency visits in the last 12 months	1.57	1.88	1.19	0.24	0.73	0.98	0.46	0.10	< 0.05
Urgent re‐admission within ≤ 30 days of previous admission									
Yes	20	11	9	0.97	16	13	3	< 0.05	0.88
No	83	46	37		63	29	34		
Admitting service									
Medical oncology	80	48	32	0.07	55	34	21	< 0.05	0.22
Radiation oncology	23	9	14		24	8	16		
Living status before hospital admission									
Independent	101	56	45	NA	78	42	36	NA	NA
At home with care	1	1	0		1	0	1		
In a rehabilitation facility	0	0	0		0	0	0		
In a long‐term care facility	0	0	0		0	0	0		
In a chronic care facility	0	0	1		0	0	0		
Direct admission to the intensive care unit									
Yes	1	0	1	NA	1	0	1	NA	0.85
No	102	57	45		78	42	36		
Survival to discharge									
Yes	86	44	42	0.05	73	37	36	0.1	0.07
No	17	13	4		6	5	1		
Urgency of admission									
Elective	45	15	30	< 0.05	73	39	34	NA	< 0.05
Emergency department visit via ambulance	14	9	5		1	1	0		
Emergency department visits without ambulance (including admissions from the clinic)	44	33	11		5	2	3		

Abbreviation: NA, not applicable.

Patients with advanced cancer may exhibit distinct characteristics compared to those acutely admitted under general medical or surgical services. The initial mHOMR study was conducted at two quaternary healthcare facilities in Toronto and included patients with cancer (20% of the cohort), however, most admissions were related to frailty (55%) or chronic organ failure (23%) [13]. Our patient cohort had several important differences: they were a young population (with a mean age of 62 years), 98.3% lived independently before admission, with a low number of emergency room visits (a mean value of one) and ambulance‐assisted admissions (mean 0.2) in the preceding 12 months—parameters all considered by the mHOMR model. Consequently, the original mHOMR score of 0.21 may be too low for this population.

Our study also explored whether the mHOMR score could be used to trigger referrals to palliative care. There were no significant differences in the proportion of mHOMR+ patients referred to palliative care at either cut‐off (58% at an mHOMR cut‐off of 0.27% vs. 55.3% at 0.21). This may represent local practices; our center has a robust and well‐integrated palliative care team with already high referral rates at the time of the study. This outcome may be better explored in a newly developed service or in a setting where referral rates are low. An ideal flagging system would combine both clinical expertise and predictive algorithms to identify high‐risk patients. It should strike a balance, not overloading resources but uncovering individuals with elevated risk of death who might otherwise be overlooked by clinical teams [16]. In this regard, automated mortality prediction models may be useful in overcoming some traditional barriers to palliative care referral including prognostic uncertainty and clinician referral variability [17, 18, 19, 20], and provide more equitable access to palliative care services. In this study, we also found a group of patients with low mHOMR scores who were referred to palliative care based on clinical judgment, and who subsequently died within 12 months, which serves as an important reminder that automated tools should be used to complement or augment, rather than replace clinical judgment.

Our findings highlight the risk that an inappropriately low mHOMR score may identify larger numbers of patients that can be supported by palliative care teams, and the importance of considering modifying the trigger score based both on the target patient population, as well as local palliative care resources. Where access to palliative care services may be limited, mHOMR scores may be used instead to prompt educational initiatives for oncologists and hospitalists to allow them to provide high‐quality secondary palliative care, including symptom management and advance care planning, or be used to advocate for additional specialist services at the organization level. There may be additional challenges associated with implementing an automated tool within a hospital's electronic medical system, such as implementation cost or the risk of alert fatigue, which will be site‐ and service‐dependent [14]. Further prospective research is needed to evaluate the benefits and challenges of this approach, to understand reasons for discrepancies between patients flagged by mHOMR and by clinicians, and to identify the most effective ways to implement and optimize such systems alongside clinical practice.

Our study has several limitations. These include the retrospective design with data extracted from the electronic health record, which may not include all relevant information such as detailed data on symptom burden or a declined referral to palliative care. There were missing data regarding the place of death for patients who died outside of our institution, and we were unable to collect data on race or socio‐economic status. The context of our study, a single quaternary‐level cancer center with a comprehensive palliative care service, also limits its generalizability. Our study is an association study, with a limited sample size; larger predictive studies with an external validation set are necessary to validate the robustness of alternative mHOMR score thresholds, which should be compared using Receiver Operating Characteristics and precision‐recall curves. As such, the mHOMR cut‐off of 0.27 for our study cannot be recommended or generalized to other study populations without further validation.

## 5 Conclusions

In this study, the developer‐recommended mHOMR cut‐off of 0.21 failed to identify a significant risk of death within 12 months among inpatients with advanced cancer. Applying a higher cut‐off of 0.27 based on the cohort median was associated with a significant risk of death within 12 months and may represent a superior cut‐off for this patient population. While mHOMR may be a useful tool to use alongside clinical decision‐making to prompt a referral to palliative care for patients with advanced cancer, the optimal cut‐off warrants further exploration.

## Author Contributions

**A. Ghoshal:** data curation (equal), formal analysis (equal), investigation (equal), methodology (equal), software (equal), visualization (equal), writing – original draft (equal), writing – review and editing (equal). **R. Prince:** conceptualization (equal), investigation (equal), methodology (equal), writing – review and editing (equal). **J. Downar:** methodology (equal), writing – review and editing (equal). **J. Lapenskie:** methodology (equal), writing – review and editing (equal). **S. Subramaniam:** methodology (equal), writing – review and editing (equal). **P. Wegier:** methodology (equal), writing – review and editing (equal). **L. W. Le:** formal analysis (equal), methodology (equal), software (equal), validation (equal), visualization (equal), writing – review and editing (equal). **B. Hannon:** conceptualization (equal), data curation (equal), investigation (equal), methodology (equal), project administration (equal), resources (equal), supervision (equal), visualization (equal), writing – original draft (equal), writing – review and editing (equal).

## Ethics Statement

The University Health Network Research Ethics Board approved the study. Written informed consent was not obtained from the participants as the study was based on routinely collected data from charts.

## Conflicts of Interest

The authors declare no conflicts of interest.

## Data Availability

Data are available from the corresponding author on request.
